# The Effect of Hemolysis on Biochemical Tests: Determining Interference Cutoffs and Managing Hemolytic Samples

**DOI:** 10.1155/jamc/9307894

**Published:** 2026-02-26

**Authors:** Babak Shirazi Yeganeh, Neda Soleimani, Saeedeh Zare, Sahand Mohammadzadeh, Farzaneh Amiri, Mohammad Javad Esmaeili, Davoud Soleimani, Soodabeh Khoshnyat

**Affiliations:** ^1^ Department of Pathology, Shiraz Medical School, Shiraz University of Medical Sciences, Shiraz, Iran, sums.ac.ir; ^2^ Department of Pathology, Shiraz Transplant Center, Abu Ali Sina Hospital, Shiraz University of Medical Sciences, Shiraz, Iran, sums.ac.ir; ^3^ Center of Policy Planning, Shiraz University of Medical Sciences, Shiraz, Iran, sums.ac.ir

**Keywords:** biochemistry, hemolysis, hemolytic index, interference

## Abstract

**Background:**

Hemolysis is a common source of interference in biochemical tests, potentially leading to significant errors in clinical decision‐making and patient management. This study aimed to evaluate the impact of varying degrees of hemolysis on routine biochemical analytes and to find a local hemolytic index (HIX) cutoff for safe reporting.

**Methods:**

This experimental study was conducted on 30 serum samples from healthy individuals with normal biochemical profiles. Baseline concentrations of 20 routine analytes were measured using a DIRUI CS‐1200 analyzer. To simulate hemolysis, increasing concentrations of autologous hemolysate were added to each sample, creating three grades of hemolysis based on HIX (mild, moderate, and severe). All analytes were remeasured, and the absolute and relative biases were calculated against baseline levels. These biases were then compared with the CLIA‐defined total allowable error (TEa) to obtain the safe reporting cutoff for each analyte.

**Results:**

All analytes showed a positive bias with increasing hemolysis, though the magnitude of interference varied significantly. LDH, uric acid, AST, and total bilirubin, in that order, were more impacted by hemolysis. In mild hemolysis (HIX < 0.5), none of the analyte biases exceeded TEa limits (*p* value < 0.05). In moderate and severe hemolysis, however, most exceeded acceptable limits. Based on these results, a HIX cutoff of 0.5 was regarded for reporting mildly hemolyzed samples.

**Conclusion:**

Hemolysis interference is both analyte‐ and system‐dependent. Laboratories are recommended to conduct similar research tailored to their local laboratory settings and population to improve analytical quality and reduce unnecessary specimen rejection.

## 1. Introduction

Exogenous and endogenous substances can impact the results of a chemical assay. The most common and significant endogenous interferers are cell‐free hemoglobin, lipids, bilirubin, autoantibodies, and heterophile antibodies. The presence of one or more of these substances in a serum or plasma sample can bias the results, producing inaccurate interpretations [1, 2].

Cell‐free hemoglobin, also known as hemolysis, is defined as the rupture of the surrounding red blood cell (RBC) membranes, followed by the release of intracellular components into serum or plasma [3]. Hemolysis can occur both in vivo and in vitro. In vivo hemolysis can occur before blood collection due to pathological conditions such as infection, immune‐mediated processes, genetic diseases of RBCs, or disseminated intravascular coagulation (DIC). Only 2%‐3% of all hemolyzed samples contain in vivo hemolysis; on the other hand, in vitro hemolysis is the most common preanalytical error, accounting for over 60% of clinical chemistry sample rejections worldwide [4–9]. Increased RBC fragility, errors committed during the phlebotomy procedure, and improper specimen preparation and transportation are the most common causes of hemolysis observed in laboratory medicine [10, 11].

Sample rejection in hemolysis has a significant financial impact due to the need to repeat hemolyzed samples, longer turnaround times (TAT) as a result of sample rejection, diagnostic mistakes, unsuitable follow‐up tests or therapies, and longer hospital stays [10, 12]. Furthermore, if in vivo hemolysis is verified, rejecting samples is not advised. Such samples should be accepted for examination and, if possible, reported, as the results are accurate representations of the contents of the body [9, 10].

Most standard chemical measurements are impacted by hemolysis through four mechanisms. First, hemolysis may falsely raise blood components, such as alanine aminotransferase (ALT), aspartate aminotransferase (AST), blood urea nitrogen (BUN), lactate dehydrogenase (LDH), magnesium, phosphate, and potassium, as these substances are highly concentrated in RBCs and are released during RBC lysis. Second, colorimetric and turbidimetric values for other chemicals, such as bilirubin, may be mistakenly increased or decreased by free hemoglobin produced during hemolysis because hemoglobin absorbs heavily at 415, 540, and 570 nm. Third, because of the competitive activity of RBC adenylate kinase, hemolysis may unintentionally suppress certain metabolic events, such as the creatine kinase (CK) activity, and the fourth is sample dilution, which can have an impact on all other analytes. The ultimate effect is determined by the sum of the effects of several mechanisms [4, 13–16].

When the amount of cell‐free hemoglobin grows, serum or plasma’s color changes from orange at the hemolysis cutoff of 0.5 g/L to red and finally dark brown (10–20 g/L). Compared to automated methods (spectrophotometry) for the assessment of serum or plasma hemolysis, which are fast, accurate, automated, and reasonably priced, visual inspection is not only inaccurate but also highly variable across observers. Furthermore, automated measurement improves the identification of slightly hemolyzed materials that are not visually identifiable or are misjudged by laboratory staff [4, 9, 17].

Owing to the significance of the degree of hemolysis and to avoid needless sample rejection, it is essential to create and coordinate suitable techniques to determine potential cutoff points for the appropriate management of hemolysis. The hemolytic impact is largely dependent on the method and instrument, and different levels of hemolysis, as measured by the hemolytic index (HIX), are correlated with different levels of interference [10, 18–23].

Although cutoff points for rejection of samples are commonly recommended by the manufacturers of analytical systems, results of end‐user verification frequently diverge from what the manufacturer says. The Clinical and Laboratory Standards Institute (CLSI) recommends that the laboratories verify the intended usefulness, strengths, and limitations of manufacturer‐derived cutoff points before they are implemented [16, 21–25].

This study aimed to evaluate the effects of hemolysis at various HIX levels in our biochemical laboratory and establish a protocol for the management of hemolytic samples.

## 2. Methods

### 2.1. Study Design and Ethical Considerations

This cross‐sectional study was carried out in January 2024 at the clinical chemistry laboratory of Nemazee and Abu Ali Sina Hospitals (Shiraz, Iran), which are general and transplantation facilities, respectively. The study adhered to the principles of the Declaration of Helsinki. Moreover, the protocol was reviewed and approved by the Ethics Committee of Shiraz University of Medical Sciences (IR.SUMS.MED.REC.1401.555). The approval includes the use of fully de‐identified leftover clinical specimens, which are exempt from informed consent under institutional regulations.

### 2.2. Sample Preparation and Baseline Testing

Thirty leftover serum samples from healthy individuals with normal biochemical results were collected. Samples exhibiting initial biochemistry abnormalities or preanalytical issues (visible hemolysis, icterus, or lipemia) were excluded. All baseline measurements were performed using a DIRUI CS‐1200 autoanalyzer (DIRUI Industrial Co., China) for 20 routine biochemical analytes (including albumin, ALT, alkaline phosphatase [ALP], AST, bilirubin direct, bilirubin total, BUN, calcium, cholesterol total, creatinine, glucose, iron, LDH, magnesium, phosphorus, potassium, protein total, sodium, triglyceride, and uric acid), and the results were used as the reference/baseline values for subsequent analysis. The DIRUI CS‐1200 chemistry autoanalyzer used in this study was installed in our laboratory in 2019. All of the reagents were of analytical grade, and two levels of analysis were performed on internal quality control (QC) materials: level 1 (normal) and level 2 (high). Table 1 shows the details of the reagents.

**TABLE 1:** Characteristics of reagents used in the study.

Analyte	Reagent	Method	Wavelength (nm)	Kit‐stated cutoff for free hemoglobin (g/L)	CLIA allowable total error
Albumin	Biorex	BCG	546	4.0	10%
ALT	Biorex	UV.IFCC	340	4.0	20%
ALP	Biorex	DGKC	405	1.5	30%
AST	Biorex	UV.IFCC	340	1.0	20%
Bilirubin total	Biorex	Photometric 2,4‐dichloroanyline	546	0.4	20%
Bilirubin direct	Biorex	Photometric 2,4‐dichloroanyline	546	0.4	20%
BUN	Biorex	Urease	340	4.0	9%
Calcium	Biorex	CPC	620	4.0	1 mg/dL
Cholesterol total	Biorex	Enzymatic	505	2.0	10%
Creatinine	Biorex	Jaffe	500	2.0	15%
Glucose	Biorex	GOD‐PAP	500	7.5	10%
Iron	Biorex	Photometric, Ferene	600	0.8	20%
LDH	Biorex	Enzymatic	340	^∗^	20%
Magnesium	Biorex	Colorimetric	546	^∗^	25%
Phosphorus	Biorex	Phosphomolybdate	340	5.0	10%
Potassium	—	ISE	—	NA	0.5 meq/L
Protein total	Biorex	Biuret	546	4.0	10%
Sodium	—	ISE	—	NA	4 meq/L
Triglyceride	Biorex	Enzymatic	505	6.2	25%
Uric acid	Biorex	Enzymatic	555	0.4	17%

Abbreviations: ALP, alkaline phosphatase; ALT, alanine aminotransferase; AST, aspartate aminotransferase; BCG, bromocresol green; BUN, blood urea nitrogen; CPC, cresol phthalein complexone; DGKC, German Society of Clinical Chemistry; GOD‐PAP, glucose oxidase/peroxidase; IFCC, International Federation of Clinical Chemistry; ISE, ion selective electrode; LDH, lactate dehydrogenase.

^∗^Hemolysis‐sensitive; no defined threshold.

### 2.3. Hemolysate Preparation and HIX Setting

Hemolysate was prepared for each case by mixing the heparinized whole blood of the cases with distilled water in a 1:1 ratio. The lysate was centrifuged at 3000 g for 10 min, and the supernatant was separated. The HIX was determined spectrophotometrically at 571 nm using a saline‐based blanking method [19, 26]. The free hemoglobin concentrations of hemolysates were used for calibration.

### 2.4. Simulation of Hemolysis and Interference Assessment

Increasing volumes of hemolysate were added to aliquots of each serum sample to obtain three defined levels of hemolysis based on HIX levels (g/L): mild (< 0.5 g/L), moderate (0.5–3.0 g/L), and severe (> 3.0 g/L), based on published recommendations by the IFCC and Guder et al. and adapted to the characteristics of the DIRUI system [27]. Subsequently, each new sample was reanalyzed for the same analytes under identical conditions. The dilution factor was accounted for result correction.

### 2.5. Bias Calculation and Interference Estimation

For each analyte, the absolute and relative differences (represented as bias) were calculated by comparing hemolyzed and baseline values. The obtained biases were compared against the CLIA‐defined total allowable error (TEa) to determine the safe reporting threshold [28]. The samples with biases greater than TEa were considered significant and nonreportable.

### 2.6. Statistical Analysis

SPSS v. 25.0 software (IBM Corp., Armonk, NY, USA) was used to analyze all the data. Descriptive statistics, including mean, standard deviation (SD), and percentage, were employed. Differences between hemolysis levels (mild, moderate, and severe) and the baselines were analyzed using repeated‐measures ANOVA with post hoc pairwise comparisons adjusted using the Bonferroni method. The significance threshold for the two‐sided test was less than 0.05.

## 3. Results

A total of 30 serum samples were evaluated at baseline and in three hemolytic states (mild, moderate, and severe). The mean HIX values for each state were 0.11, 0.69, 1.6, and 3.4 gr/L, respectively (Table 2).

**TABLE 2:** The results of HIX and biochemistry analytes in baseline samples and their hemolytic counterparts.

Analyte (unit)	Mean ± SD			
	Baseline	Mild hemolysis	Moderate hemolysis	Severe hemolysis
HIX (g/L)	0.11 ± 0.12	0.69 ± 0.24	1.6 ± 0.54	3.4 ± 1.10
Albumin (gr/dL)	4.7 ± 0.3	4.9 ± 0.3	10.1 ± 0.6	15.7 ± 1.3
AST (IU/L)	24 ± 7	26 ± 7	63 ± 14	131 ± 30
ALT (IU/L)	24 ± 9	25 ± 9	54 ± 20	74 ± 26
ALP (IU/L)	180 ± 39	184 ± 40	349 ± 76	391 ± 110
BUN (mg/dL)	12 ± 2	13 ± 3	24 ± 5	31 ± 7
Bilirubin direct (mg/dL)	0.26 ± 0.11	0.27 ± 12	0.52 ± 0.25	0.58 ± 0.19
Bilirubin total (mg/dL)	0.63 ± 0.23	0.71 ± 0.27	1.57 ± 0.54	2.46 ± 0.63
Calcium (mg/dL)	10.4 ± 0.3	10.7 ± 0.5	21.7 ± 0.9	29.2 ± 1.9
Cholesterol total (mg/dL)	185 ± 33	190 ± 35	376 ± 70	489 ± 93
Creatinine (mg/dL)	0.80 ± 0.11	0.83 ± 1.46	1.43 ± 0.29	1.87 ± 0.35
Glucose (mg/dL)	97 ± 8	101 ± 10	203 ± 17	272 ± 23
Iron (μg/dL)	89 ± 29	91 ± 28	189 ± 64	288 ± 85
LDH (IU/L)	295 ± 38	314 ± 62	954 ± 131	2389 ± 402
Magnesium (mg/dL)	2.0 ± 0.2	2.1 ± 0.2	4.2 ± 0.3	5.6 ± 0.9
Phosphorus (mg/dL)	3.4 ± 0.5	3.6 ± 0.5	7.6 ± 1.2	11.8 ± 1.6
Potassium (meq/L)	4.2 ± 0.4	4.4 ± 0.4	9.1 ± 0.8	14.1 ± 1.2
Protein total (gr/dL)	7.5 ± 0.7	7.8 ± 0.7	15.8 ± 1.5	22.2 ± 2.2
Sodium (meq/L)	138 ± 1.7	141 ± 5.2	280 ± 18	376 ± 14
Triglyceride (mg/dL)	127 ± 78	130 ± 81	267 ± 164	348 ± 208
Uric acid (mg/dL)	5.0 ± 0.9	5.0 ± 0.7	12.6 ± 2	26 ± 6

*Note:* HIX: hemolytic index; AST: aspartate aminotransferase; ALT: alanine aminotransferase; ALP: alkaline phosphatase; LDH: lactate dehydrogenase.

Abbreviation: SD, standard deviation.

As hemolysis (HIX) increased from mild to severe, a progressive increase was observed across all analytes. Analytes that were more impacted by hemolysis were LDH, uric acid, AST, and total bilirubin, in that order; direct bilirubin was the least impacted analyte (Table 2).

In mild hemolysis (HIX < 0.5), observed biases remained within the CLIA‐defined TEa for all analytes (*p* value < 0.05), while in moderate and severe hemolysis, the majority of analytes exceeded the TEa threshold (*p* value < 0.0001) (Table 3).

**TABLE 3:** The mean differences of biochemistry results in baseline samples and their hemolytic counterparts.

Analyte	Mild hemolysis	Moderate hemolysis	Severe hemolysis
	Mean difference ± SD	Mean difference ± SD	Mean difference ± SD
Albumin (gr/dL)	0.2 ± 0.3	5.4 ± 0.6	10 ± 1
AST (IU/L)	1 ± 10	29 ± 23	49 ± 26
ALT (IU/L)	4.2 ± 49	164 ± 74	206 ± 122
ALP (IU/L)	1.5 ± 7	40 ± 18	112 ± 34
BUN (mg/dL)	0.01 ± 0.2	0.3 ± 0.3	0.2 ± 0.3
Bilirubin direct (mg/dL)	0.05 ± 0.3	0.92 ± 0.6	1.81 ± 0.6
Bilirubin total (mg/dL)	0.3 ± 2.3	12 ± 5.1	19 ± 7.3
Calcium (mg/dL)	0.2 ± 0.6	11 ± 0.8	19 ± 2
Cholesterol total (mg/dL)	3.7 ± 6	190 ± 38	304 ± 61
Creatinine (mg/dL)	0.01 ± 0.1	0.61 ± 0.3	1.04 ± 0.3
Glucose (mg/dL)	3 ± 9	105 ± 18	173 ± 23
Iron (μg/dL)	3.4 ± 40	97 ± 63	195 ± 95
LDH (IU/L)	18 ± 72	678 ± 154	2374 ± 589
Magnesium (mg/dL)	0.04 ± 0.3	2.15 ± 0.4	3.56 ± 1.1
Phosphorus (mg/dL)	0.1 ± 0.7	4.1 ± 1.1	8.3 ± 1.7
Potassium (meq/L)	0.1 ± 0.3	4.8 ± 0.8	9.9 ± 1.1
Protein total (gr/dL)	0.2 ± 0.8	8.3 ± 1.2	15 ± 2.1
Sodium (meq/L)	3.3 ± 5	142 ± 18	238 ± 14
Triglyceride (mg/dL)	2.4 ± 95	139 ± 137	221 ± 211
Uric acid (mg/dL)	0.3 ± 1	7.8 ± 2	21 ± 4.6

*Note:* HIX: hemolytic index; AST: aspartate aminotransferase; ALT: alanine aminotransferase; ALP: alkaline phosphatase; LDH: lactate dehydrogenase.

Abbreviation: SD, standard deviation.

**FIGURE 1:**
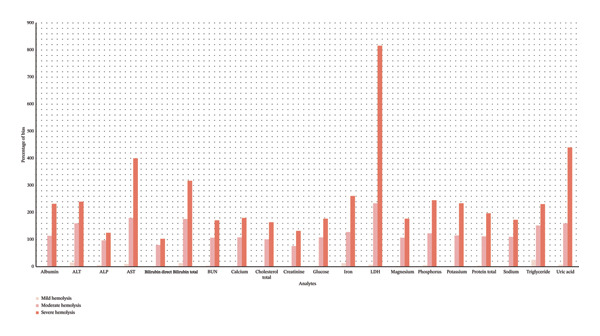
Percentage of bias for 20 biochemistry analytes at different levels of hemolysis.

**FIGURE 2:**
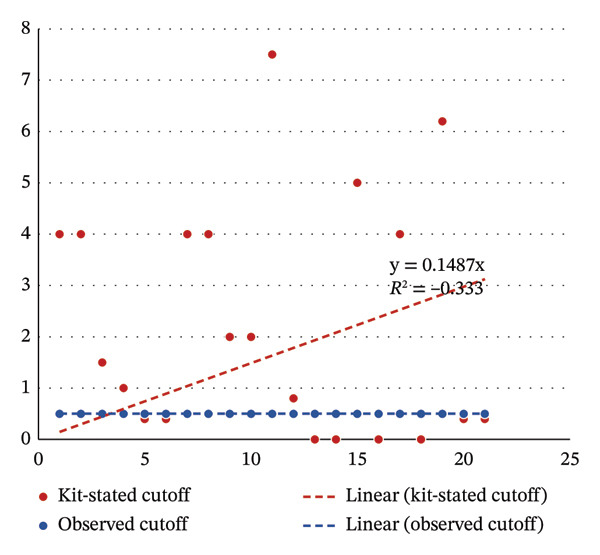
Regression analysis comparing kit‐stated hemolysis cutoffs with the locally observed cutoffs for 20 biochemical analytes.

The percentage of bias for each analyte, relative to baseline value, is illustrated in Figure 1. Nonlinear patterns of interference were especially notable in intracellular analytes, such as LDH, potassium, AST, phosphorus, and iron. Conversely, extracellular markers such as albumin and sodium showed only minor changes. As illustrated in Figure 2, manufacturer‐stated cutoffs showed wide analyte‐to‐analyte variability, whereas our observed cutoffs were uniformly distributed around 0.5 g/L.

## 4. Discussion

Hemolysis is still one of the most prevalent and inevitable interferences in clinical laboratories that has a major effect on the accuracy of biochemical test results. In this study, we investigated the effect of varying degrees of hemolysis on 20 routine biochemical analytes using the DIRUI CS‐1200 autoanalyzer.

Our findings confirmed that the degree of interference is highly analyte‐dependent and tends to follow both linear and nonlinear patterns. While all analytes revealed a positive bias with increasing HIX, the magnitude of change varied significantly. More significant alterations were seen in LDH, uric acid, AST, and total bilirubin, consistent with prior studies highlighting their high intracellular concentrations and susceptibility to hemolysis [29–31]. Moreover, the observed nonlinear positive bias for certain intracellular analytes, such as potassium and phosphorus, supports the concept of concentration‐dependent interference, which has been emphasized in recent publications [15, 32, 33]. Conversely, analytes that are mainly extracellular, including albumin, sodium, total protein, and cholesterol, show minimal or linear increases in bias, as their levels are less affected by cell lysis [34, 35].

Another notable finding was that in mild hemolysis (HIX < 0.5 g/L), the results of all analytes were below the TEa defined by CLIA, indicating acceptable analytical performance. This threshold was thus set in our laboratory as an internal reporting cutoff, facilitating a safe and practical policy for managing mildly hemolyzed samples. In cases of mild hemolysis (HIX < 0.5), results are reported alongside a clear comment indicating the presence of hemolysis and specifying the HIX value, allowing clinicians to interpret results with appropriate caution. This acceptable cutoff was significantly lower than the cutoffs provided by the reagent manufacturer for most analytes.

Importantly, our research underscores the necessity for laboratories to perform local validation of hemolysis interference rather than relying solely on manufacturer‐stated or reference‐derived cutoffs. Instruments, reagents, optical systems, and even population variations may impact hemolysis interference, and guidelines from CLSI (C56‐A) and IFCC recommend such local verifications to ensure clinical accuracy.

A key strength of our study is the comprehensive evaluation of 20 analytes under controlled hemolysis conditions, along with the application of a quantitative HIX method. Nonetheless, our results should be interpreted regarding certain limitations. First, the induced in vitro hemolysis we used may not fully represent the real in vivo hemolysis. Second, although we accounted for sample dilution, enzymatic inactivation or matrix effect could still influence the results.

## 5. Limitations

A limitation of the study is that the DIRUI CS‐1200 is a mid‐tier analyzer which, despite its use in many regional laboratories, is less commonly utilized in large tertiary or reference centers. This may reduce the generalizability of our findings to laboratories that predominantly operate Abbott, Roche, Siemens, or Beckman chemistry platforms.

Another limitation is that analyzer‐specific CV% values were not used to compute allowable bias thresholds. This omission may cause CLIA‐based limits to appear more permissive than CV‐driven criteria. Therefore, the 0.5 g/L cutoff should be considered a functional reporting threshold rather than the most conservative analytical limit. Incorporating local CV% data will strengthen future validation efforts and allow for a more stringent definition of hemolysis interference.

## 6. Conclusion

This study supports the implementation of a laboratory‐specific management protocol for hemolysis interference. The use of an HIX‐based cutoff of 0.5 gr/L allows for reliable reporting of biochemistry analytes in mild hemolysis. Other laboratories are encouraged to conduct similar research tailored to their local laboratory settings and population to improve analytical quality and reduce unnecessary specimen rejection.

## Author Contributions

N.S., S.Z., B.S.Y., and S.M. developed the concept of the study and the study design and wrote the draft manuscript. N.S., S.Z., and M.J.E. set up the tests on autoanalyzers. M.J.E. and F.A. did the analyses. N.S. and S.Z. selected the cases and samples. N.S., S.Z., D.S., and S.K. with the input of all authors interpreted the data.

## Funding

This project has no institutional funding.

## Disclosure

The views and opinions expressed in this article are those of the authors and do not necessarily reflect the official policy or position of any affiliated institutions or organizations of the authors. All authors have read and approved the final manuscript.

## Conflicts of Interest

The authors declare no conflicts of interest.

## Data Availability

The data used to support the findings of this study are included within the article.

## References

[1] Agarwal S. , Vargas G. , Nordstrom C. , Tam E. , Buffone G. J. , and Devaraj S. , Effect of Interference From Hemolysis, Icterus and Lipemia on Routine Pediatric Clinical Chemistry Assays, Clinica Chimica Acta. (2015) 438, 241–245, 10.1016/j.cca.2014.08.008, 2-s2.0-84907485552. 25128720

[2] Tian G. , Wu Y. , Jin X. et al., The Incidence Rate and Influence Factors of Hemolysis, Lipemia, Icterus in Fasting Serum Biochemistry Specimens, Public Library of Science ONE. (2022) 17, no. 1, 10.1371/journal.pone.0262748. PMC8769349 35045128

[3] Guder W. G. , Hemolysis as an Influence and Interference Factor in Clinical Chemistry, Journal of Clinical Chemistry and Clinical Biochemistry. (1986) 24, no. 2, 125–126. 3711796

[4] Simundic A. M. , Topic E. , Nikolac N. , and Lippi G. , Hemolysis Detection and Management of Hemolyzed Specimens, Biochemical Medicine. (2010) 20, 154–159, 10.11613/bm.2010.018.

[5] Jones B. A. , Calam R. R. , and Howanitz P. J. , Chemistry Specimen Acceptability: A College of American Pathologists Q-Probes Study of 453 Laboratories, Archives of Pathology and Laboratory Medicine. (1997) 121, no. 1, 19–26. 9111088

[6] Simundic A. M. , Nikolac N. , Vukasovic I. , and Vrkic N. , The Prevalence of Preanalytical Errors in a Croatian ISO 15189 Accredited Laboratory, Clinical Chemistry and Laboratory Medicine. (2010) 48, no. 7, 1009–1014, 10.1515/cclm.2010.221, 2-s2.0-77953868631. 20441481

[7] Carraro P. , Servidio G. , and Plebani M. , Hemolyzed Specimens: A Reason for Rejection or a Clinical Challenge?, Clinical Chemistry. (2000) 46, no. 2, 306–307, 10.1093/clinchem/46.2.306. 10657399

[8] Simundic A. M. , Nikolac N. , and Guder W. G. , Rifai N. , Horvath R. , and Wittwer C. , Preanalytical Variation and Preexamination Processes, Tietz Textbook of Clinical Chemistry and Molecular Diagnostics, 2018, 6th edition, Elsevier, St. Louis, Missouri, 81–120.

[9] Lippi G. , Blanckaert N. , Bonini P. et al., Hemolysis: An Overview of the Leading Cause of Unsuitable Specimens in Clinical Laboratories, Clinical Chemistry and Laboratory Medicine. (2008) 46, no. 6, 764–772, 10.1515/CCLM.2008.170, 2-s2.0-44949158707. 18601596

[10] Simundic A. M. , Baird G. , Cadamuro J. , Costelloe S. J. , and Lippi G. , Managing Hemolyzed Samples in Clinical Laboratories, Critical Reviews in Clinical Laboratory Sciences. (2020) 57, no. 1, 1–21, 10.1080/10408363.2019.1664391. 31603708

[11] Barcellini W. and Fattizzo B. , Clinical Applications of Hemolytic Markers in the Differential Diagnosis and Management of Hemolytic Anemia, Disease Markers. (2015) 2015, 635670–635677, 10.1155/2015/635670, 2-s2.0-84956931124. 26819490 PMC4706896

[12] Soleimani N. , Azadi A. , Esmaeili M. J. et al., Termination of Repeat Testing in Chemical Laboratories Based on Practice Guidelines: Examining the Effect of Rule-Based Repeat Testing in a Transplantation Center, Journal of Analytical Methods in Chemistry. (2021) 2021, 9955990–9955997, 10.1155/2021/9955990. 34055449 PMC8137285

[13] Perović A. and Dolčić M. , Influence of Hemolysis on Clinical Chemistry Parameters Determined With Beckman Coulter Tests-Detection of Clinically Significant Interference, Scandinavian Journal of Clinical and Laboratory Investigation. (2019) 79, no. 3, 154–159, 10.1080/00365513.2019.1576099, 2-s2.0-85061807212. 30767593

[14] Marques-Garcia F. , Methods for Hemolysis Interference Study in Laboratory Medicine-A Critical Review, Electronic Journal of the International Federation of Clinical Chemistry and Laboratory Medicine. (2020) 31, no. 1, 85–97. 32256292 PMC7109502

[15] Ni J. , Zhu W. , Wang Y. et al., A Reference Chart for Clinical Biochemical Tests of Hemolyzed Serum Samples, Journal of Clinical Laboratory Analysis. (2021) 35, no. 1, 10.1002/jcla.23561. PMC7843283 32881061

[16] Clinical and Laboratory Standards Institute , Hemolysis, Icterus, and Lipemia/Turbidity Indices as Indicators of Interference in Clinical Laboratory Analysis; Approved Guideline, Clinical and Laboratory Standards Institute Document C56-A. (2012) 32.

[17] Liu S. , Li J. , Ning L. , Wu D. , and Wei D. , Assessing the Influence of True Hemolysis Occurring in Patient Samples on Emergency Clinical Biochemistry Tests Results Using the VITROS 5600 Integrated System, Biomedical Reports. (2021) 15, no. 5, 10.3892/br.2021.1467. PMC8461321 34631046

[18] Du Z. H. , Liu J. Q. , Zhang H. , Bao B. H. , Zhao R. Q. , and Jin Y. , Determination of Hemolysis Index Thresholds for Biochemical Tests on Siemens Advia 2400 Chemistry Analyzer, Journal of Clinical Laboratory Analysis. (2019) 33, no. 4, 10.1002/jcla.22856, 2-s2.0-85061796687. PMC6589729 30779463

[19] Ishiguro A. , Nishioka M. , Morishige A. et al., Determination of the Optimal Wavelength of the Hemolysis Index Measurement, Journal of Clinical Medicine. (2023) 12, no. 18, 10.3390/jcm12185864. PMC10531830 37762805

[20] Nakrani M. , Musabaike W. , Mahathanthila N. Y. , and Aitkenhead H. , Haemolysed Samples: Identification and Reporting in Biochemistry Laboratory–Quality Improvement Initiative, Archives of Disease in Childhood. (2021) 106, 10.1136/archdischild-2021-gosh.69.

[21] Gidske G. , Aakre K. M. , Rustad P. et al., Handling of Hemolyzed Serum Samples in Clinical Chemistry Laboratories: the Nordic Hemolysis Project, Clinical Chemistry and Laboratory Medicine. (2019) 57, no. 11, 1699–1711, 10.1515/cclm-2019-0366, 2-s2.0-85069643066. 31617690

[22] Wan Azman W. N. , Omar J. , Koon T. S. , and Tuan Ismail T. S. , Hemolyzed Specimens: Major Challenge for Identifying and Rejecting Specimens in Clinical Laboratories, Oman Medical Journal. (2019) 34, no. 2, 94–98, 10.5001/omj.2019.19, 2-s2.0-85065171920. 30918601 PMC6425048

[23] Getahun T. , Alemu A. , Mulugeta F. et al., Evaluation of Visual Serum Indices Measurements and Potential False Result Risks in Routine Clinical Chemistry Tests in Addis Ababa, Ethiopia, Electronic Journal of the International Federation of Clinical Chemistry and Laboratory Medicine. (2019) 30, no. 3, 276–287. 31695585 PMC6803774

[24] Erkal F. A. , Aykal G. , Yalçınkaya H. M. , Aksoy N. , and Özdemir M. , The Effect of Automated Hemolysis Index Measurement on Sample and Test Rejection Rates, Turkish Journal of Biochemistry. (2019) 44, no. 5, 630–634, 10.1515/tjb-2018-0462.

[25] Monneret D. , Mestari F. , Atlan G. et al., Hemolysis Indexes for Biochemical Tests and Immunoassays on Roche Analyzers: Determination of Allowable Interference Limits According to Different Calculation Methods, Scandinavian Journal of Clinical and Laboratory Investigation. (2015) 75, no. 2, 162–169, 10.3109/00365513.2014.993691, 2-s2.0-84924303067. 25608598

[26] Xie H. , Wei J. , and Luo X. , A Self-Established Method for the Quantitative Determination of Plasma Free Hemoglobin Utilizing the Hemolysis Index of Siemens ADVIA 2400 Chemistry System, Journal of Laboratory Physicians. (2024) 16, 582–586, 10.25259/JLP_130_2024.

[27] Guder W. G. , Narayanan S. , Wisser H. , and Zawta B. , The Impact of Preanalytical Variables on the Quality of Laboratory Results, 2009, 3rd edition, Wiley-Blackwell.

[28] Clinical Laboratory Improvements Amendments of 1988 , Final Rule. Laboratory Requirements, Federal Register. (1992) 57, 7002–7288. 10170937

[29] Parambu M. M. and Bush V. , Evaluation of Sensitive Analytes to Hemolysis Interference on an Automated Chemistry Analyzer, Journal of Applied Laboratory Medicine. (2024) 9, no. 3, 558–564, 10.1093/jalm/jfad124. 38300631

[30] Lippi G. , Salvagno G. L. , Montagnana M. , Brocco G. , and Guidi G. C. , Influence of Hemolysis on Routine Clinical Chemistry Testing, Clinical Chemistry and Laboratory Medicine. (2006) 44, no. 3, 311–316. 16519604 10.1515/CCLM.2006.054

[31] Lippi G. and Plebani M. , Hemolysis: An Overview of the Leading Cause of Unsuitable Specimens in Clinical Laboratories, Clinical Chemistry and Laboratory Medicine. (2012) 50, no. 5, 775–782, 10.1515/cclm-2011-0887. 18601596

[32] Tang N. Y. , Mitchell K. R. , Groboske S. E. et al., Reducing Specimen Rejection Rates Using Concentration-Dependent Hemolysis Rejection Thresholds, Journal of Applied Laboratory Medicine. (2023) 8, no. 2, 285–295, 10.1093/jalm/jfac095. 36592084

[33] Rosemark C. L. , Baumann N. , Block D. , and Andress B. , Reducing Hemolyzed Specimen Rejection for Aspartate Aminotransferase (AST): A Quality Improvement Initiative to Further Optimize Concentration-Specific H-index Thresholds, Clinical Chemistry. (2024) 70, no. 1, hvae106–hvae197, 10.1093/clinchem/hvae106.097.

[34] Koseoglu M. , Hur A. , Atay A. , and Cuhadar S. , Effects of Hemolysis Interferences on Routine Biochemistry Parameters, Biochemical Medicine. (2011) 21, no. 1, 79–85, 10.11613/bm.2011.015. 22141211

[35] Yanagisawa Y. , Isobe K. , Naito A. et al., Influence of in Vitro Hemolysis on 80 Different Laboratory Tests, Clinical Laboratory. (2017) 63, no. 02/2017, 219–226, 10.7754/clin.lab.2016.160305, 2-s2.0-85016279445. 28182357

